# Midventricular Hypertrophic Cardiomyopathy with Apical Aneurysm: Potential for Underdiagnosis and Value of Multimodality Imaging

**DOI:** 10.1155/2016/9717948

**Published:** 2016-01-20

**Authors:** Archana Sivanandam, Karthik Ananthasubramaniam

**Affiliations:** ^1^University of California, Los Angeles, 405 Hilgard Avenue, Los Angeles, CA 90024, USA; ^2^Heart and Vascular Institute, Henry Ford Hospital, 2799 W. Grand Boulevard, Detroit, MI 48322, USA

## Abstract

We illustrate a case of midventricle obstructive HCM and apical aneurysm diagnosed with appropriate use of multimodality imaging. A 75-year-old African American woman presented with a 3-day history of chest pain and dyspnea with elevated troponins. Her electrocardiogram showed sinus rhythm, left atrial enlargement, left ventricular hypertrophy, prolonged QT, and occasional ectopy. After medical therapy optimization, she underwent coronary angiography for an initial diagnosis of non-ST segment elevation myocardial infarction. Her coronaries were unremarkable for significant disease but her left ventriculogram showed hyperdynamic contractility of the midportion of the ventricle along with a large dyskinetic aneurysmal apical sac. A subsequent transthoracic echocardiogram provided poor visualization of the apical region of the ventricle but contrast enhancement identified an aneurysmal pouch distal to the midventricular obstruction. To further clarify the diagnosis, cardiac magnetic resonance imaging with contrast was performed confirming the diagnosis of midventricular hypertrophic cardiomyopathy with apical aneurysm and fibrosis consistent with apical scar on delayed enhancement. The patient was medically treated and subsequently underwent elective implantable defibrillator placement in the ensuing months for recurrent nonsustained ventricular tachycardia and was initiated on prophylactic oral anticoagulation with warfarin for thromboembolic risk reduction.

## 1. Introduction

Hypertrophic cardiomyopathy (HCM) is an inherited disorder of the cardiac muscle and is well known as the most common cause of sudden cardiac death in individuals less than 35 years of age in North America. It is an autosomal dominant condition with reported prevalence of 1 in 500. HCM is caused by a genetic defect which results in the mutation of the sarcomere or its associated proteins resulting in dysfunction of the myocardium. Most HCM patients (90%) are diagnosed with asymmetric septal hypertrophy, with the less common variations being midventricular/apical (1%) and posteroseptal and isolated lateral wall hypertrophy (1%). Although only 25–35% of the patients demonstrate obstruction at rest, provocable gradients can be demonstrated in up to 75% of HCM patients [1].

Pathophysiology of HCM includes left ventricular outflow tract obstruction, diastolic dysfunction, myocardial ischemia, autonomic dysfunction, and mitral valve regurgitation [2].

We present a case where initial diagnosis of apical ballooning (Takotsubo cardiomyopathy) was suspected at coronary angiography, but subsequent multimodality imaging with echocardiography and magnetic resonance established the unique entity of midventricular HCM with apical aneurysm.

## 2. Case Report

**Figure 1:**
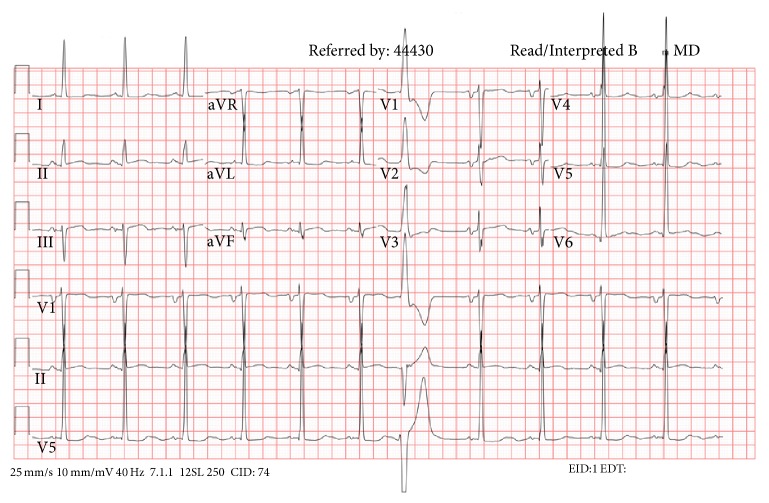
12-lead electrocardiogram.

A 75-year-old African American female with stage 4 chronic kidney disease presented with a 3-day history of atypical chest pain and dyspnea. She had a long standing history of hypertension. At presentation, she was hypertensive with blood pressure of 180/100 mm Hg. The electrocardiogram ordered showed sinus rhythm (heart rate: 85 beats/min), ST depression, and deep T wave abnormality (Figure 1). Initial troponin I level was 1.5 ng/mL, and she was diagnosed with non-ST segment elevation myocardial infarction. She was started on aspirin, clopidogrel, heparin, and beta-blockers and underwent coronary angiography. The angiogram showed nonobstructive mild coronary artery disease.

**Figure 2:**
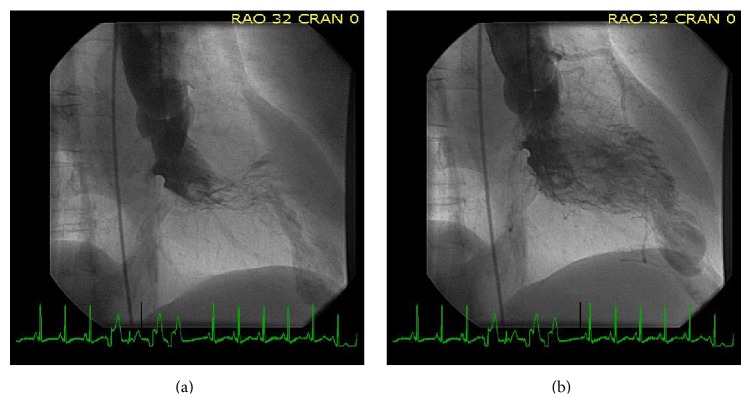
Left ventriculogram demonstrating midventricular obstruction with large aneurysmal apical pouch (diastole and systole).

Left ventriculogram was done and showed a hyperdynamic midportion of the ventricle with a large aneurysmal dyskinetic sac with stasis of contrast (Figures 2(a) and 2(b)). Given her acute coronary syndrome-like presentation, it was suspected that she had possible Takotsubo cardiomyopathy (apical ballooning) and was admitted for further evaluation.

**Figure 3:**
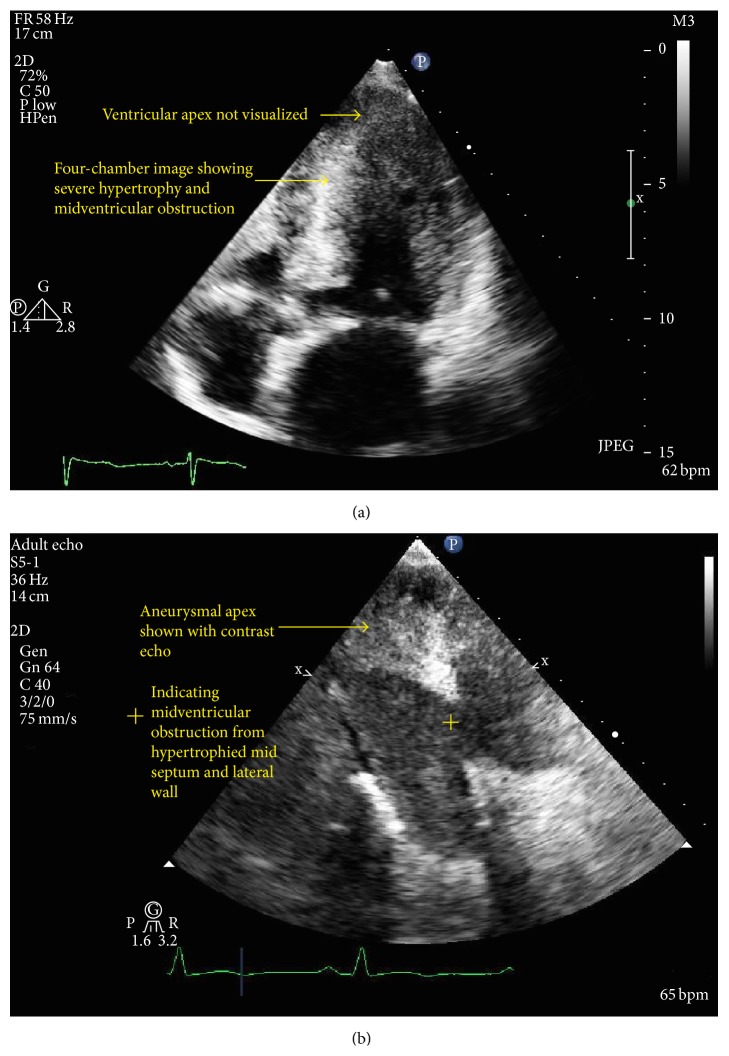
(a) Noncontrast transthoracic echocardiographic 4-chamber image demonstrating left ventricular hypertrophy and inability to visualize apical pouch. (b) Contrast transthoracic echocardiographic image demonstrating midventricular narrowing with aneurysmal apex.

**Figure 4:**
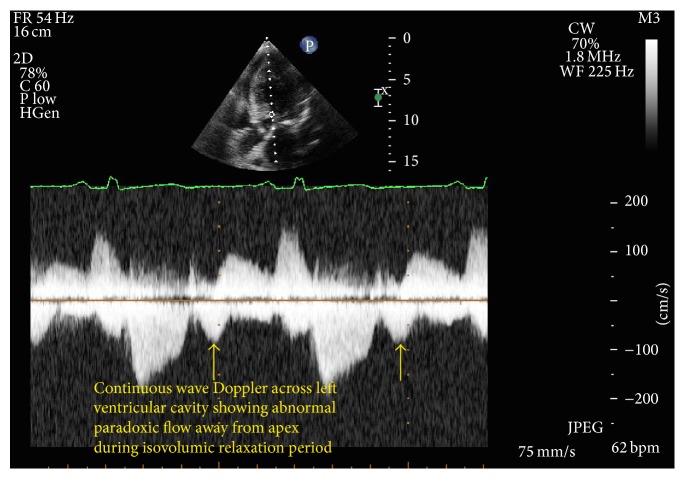
Doppler flow pattern across midventricular obstructive ventricle.

She underwent transthoracic echocardiogram the next day, and the noncontrast image shown in Figure 3(a) displayed moderate left ventricular hypertrophy including asymmetric septal hypertrophy but poor visualization of the apex and no definite dyskinetic cavity as shown on the left ventricular angiogram. However, to define the apex better, echocardiographic contrast was utilized and the contrast images clearly demonstrated midventricular narrowing, a hyperdynamic zone, and apical aneurysmal zone similar to coronary angiogram (Figure 3(b)). There was flow acceleration demonstrable with paradoxic Doppler flow between the mid cavity narrowing and the apical aneurysmal zone (Figure 4).

**Figure 5:**
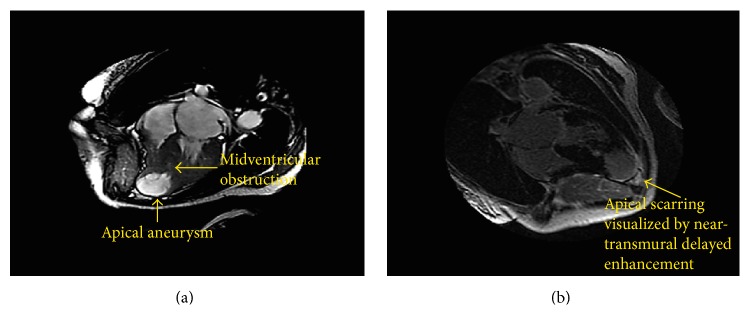
(a) Cine 4-chamber image of cardiac MRI done with steady state free precession imaging (SSFP) demonstrating diffuse hypertrophy with aneurysmal apex. (b) Delayed enhancement 4-chamber MRI image demonstrating thinned apex and apical scarring.

Based on the constellation of findings, she was diagnosed with midventricular HCM with apical aneurysm formation. She underwent cardiac magnetic resonance imaging (MRI) with gadolinium contrast that confirmed the diagnosis and further illustrated concomitant outflow tract obstruction and significant septal hypertrophy with moderate to severe secondary mitral regurgitation (Figures 5(a) and 5(b)). During her hospital course, she had multiple bouts of nonsustained ventricular tachycardia. She subsequently underwent implantable cardioverter defibrillator placement and was initiated on warfarin for stroke prevention.

## 3. Discussion

This case represents an uncommon variant of HCM, namely, midventricular obstructive variant with apical aneurysm formation, which occurs in only 1-2% of HCM patients [1]. Although a different diagnosis was entertained initially, strengths of multimodality imaging performed appropriately in this case clinched the final diagnosis and significantly impacted patient management.

The pathophysiology of apical aneurysm formation is fascinating. The midventricular variant of HCM involves hypertrophy of the midventricle, which can be exacerbated by hypertension. The hypertrophy creates 2 adjacent cavities on either side of the obstruction during systole. The proximal cavity develops a low pressure zone and the distal cavity forms a high pressure zone leading to necrosis due to chronic subendocardial ischemia. This subsequently leads to scarring, thinning, and apical aneurysm formation in the infarcted tissue [1].

Apical aneurysm formation and the decrease in cardiac output are attributed to a higher risk of small vessel disease and increase the chances for sudden cardiac arrest or acute myocardial infarction from ventricular arrythmias and thrombus formation due to stasis of blood [3, 4].

Of note, noncontrast echocardiography did not correctly delineate this apical aneurysmal zone well. It is well known that foreshortening of the apex is a limitation of transthoracic echocardiograms. However, the addition of contrast imaging highlighted the apical dyssynergic zone very well. Unique Doppler patterns first described in midventricular HCM by Nakamura et al. (paradoxic jet flow) were also noted in our patient with Doppler echocardiography [5]. These unique Doppler flow patterns are often the clue towards concealed apical asynergy/dyssynergy, as was seen in our case. The paradoxic jet flow is from the apex to the base and is likely related to higher diastolic pressure at the apex moving blood to the lower pressure proximal zone, and in the original description by Nakamura et al., it was observed in 20/198 patients with midventricular HCM. Interestingly, such patients were found to have higher incidence of systemic embolism, ventricular arrhythmias, and thallium perfusion defects in their study. It is possible that persistent high diastolic pressure compromises subendocardial perfusion and contributes to apical necrosis and the development of aneurysmal deformation. Importantly, as Doppler is independent of image quality, such unique Doppler patterns can alert clinicians of apical pouch/midventricular obstructive physiology.

Cardiac MRI carries unique value in the workup of HCM as it helps to detect more atypical variants of HCM such as apical HCM and anterolateral wall variant HCM, which seems to be underrecognized by echocardiography due to limitations in adequately imaging these areas.

Cardiac MRI is also recommended as an adjunctive test by the 2011 guidelines for evaluating the anatomy when the decision regarding septal ablation versus myectomy is not clear [2]. Mitral valve morphologic variations, anomalous location of papillary muscles, multiple papillary muscle heads, and myocardial crypts are numerous additional findings that can be better demonstrated by cardiac MRI. More recently, cardiac MRI with delayed enhancement imaging with gadolinium has been shown to have increasing prognostic value with regard to assessment of myocardial scarring, which is noted in a majority of patients with HCM. In our patient, myocardial scarring was demonstrated in the infarcted apical zone, and such areas increase the risk for ventricular arrhythmias. Recent data suggest that the extent of scarring demonstrated on cardiac MRI greater than or equal to 15% of the total myocardium may be associated with an increased risk of cardiac events in HCM patients [6].

Treatment of midventricular HCM is targeted at reducing the symptoms, such as the intraventricular gradient, and the risk of complications, such as heart failure and sudden cardiac death. Beta-blockers and calcium blockers can contribute to reduction of obstructive gradients with negative inotropic effect, decreasing outflow obstruction and restoring cardiac output [2]. Septal myectomy, through resection of a portion of the septum, can achieve a similar effect by widening the outflow tract if concomitant outflow obstruction is present but may not be applicable to the midventricular variant [1].

For patients with midventricular HCM and apical aneurysm, treatment options are controversial. Relief of obstruction at the mid- and basal ventricle follows standard recommendations as in the guidelines for HCM [2, 7], but this subset of patients is at a higher risk for ventricular arrhythmias and has been identified as one of the anatomic substrates in decision-making towards a primary prevention implantable cardioverter defibrillator (ICD). Anticoagulants are not normally given to patients with traditional HCM unless concomitant atrial fibrillation is noted; however, the midventricular HCM with apical aneurysm variant may represent a higher risk variant due to apical stasis of the blood and high risk of thrombus formation and resulting embolization and warrant anticoagulation [2].

## 4. Conclusion

This report outlines a clinical presentation of obstructive midventricular HCM with apical aneurysm formation as identified by multimodality imaging. Clinicians should be aware of the manifestations of this entity to avoid misdiagnosis or underdiagnosis, to use multimodality imaging appropriately to delineate the anatomic substrate, to recognize the challenges in management of these patients, and to consider prophylactic anticoagulation and implantable cardioverter defibrillator placement to decrease adverse cardiac events in this subset.
